# Farm Exposure as a Differential Risk Factor in ANCA-Associated Vasculitis

**DOI:** 10.1371/journal.pone.0137196

**Published:** 2015-09-04

**Authors:** P. Willeke, B. Schlüter, C. Sauerland, H. Becker, S. Reuter, A. Jacobi, H. Schotte

**Affiliations:** 1 Department of Medicine D, Section of Rheumatology and Clinical Immunology, University Hospital Münster, Münster, Germany; 2 Centre for Laboratory Medicine, University Hospital Münster, Münster, Germany; 3 Institute of Biostatistics and Clinical Research, University of Münster, Münster, Germany; 4 Division of Rheumatology and Clinical Immunology, Brandenburg Medical School, Neuruppin, Germany; Robert Bosch Hospital, GERMANY

## Abstract

**Objective:**

To investigate the association of farm exposure and the development of ANCA-associated vasculitis (AAV).

**Methods:**

One hundred eighty-nine well defined patients with AAV (n = 119 with granulomatosis with polyangiitis [GPA], n = 48 with microscopic polyangiitis [MPA], n = 22 patients with eosinophilic granulomatosis with polyangiitis [EGPA]) and 190 controls (n = 119 patients with rheumatoid arthritis, n = 71 with large vessel vasculitis) were interrogated using a structured questionnaire. Factors investigated were occupation, farm exposure, contact to different livestock, participation in harvesting, residence next to a farm, MRSA status, and contact to domestic pets at disease onset or ever before. The odds ratio (OR) and 95% confidence interval [95%CI] were calculated for each item.

**Results:**

Univariate analysis revealed a strong association of AAV with regular farm exposure; OR 3.44 [95%CI 1.43–8.27]. AAV was also associated with regular contact to cattle 4.30 (1.43–8.27), pigs 2.75 (1.12–6.75) and MRSA carriage 3.38 (1.11–10.3). This association was stronger in the subgroup of GPA patients. OR in this group for farm exposure was 4.97; [2.02–12.2], for cattle 6.71 [95% CI 2.19–20.7], for pigs 4.34 [1.75–10.9], and MRSA carriage 5.06 [1.62–15.8]). There was no significant association of MPA or EGPA with these parameters.

**Conclusion:**

A significant association between farm exposure or farm animal exposure and AAV especially in the subgroup of patients with GPA has been identified. This suggests that these entities are distinct and have different triggers for the immune process.

## Introduction

Antineutrophil cytoplasmic antibody (ANCA)-associated vasculitis (AAV) is a systemic necrotising vasculitis predominantly affecting small vessels. AAV comprises granulomatosis with polyangiits (GPA, Wegener’s), microscopic polyangiitis (MPA), and eosinophilic granulomatosis with polyangiitis (EGPA) [1]. The annual incidence of AAV as a group is estimated at about 10–20 patients per million [2]. The causes of AAV are poorly understood and their pathophysiological mechanisms remain uncertain [3].

In a recent genome-wide association study of patients with AAV a genetic contribution to the pathogenesis has been confirmed [4]. Beside the genetic susceptibility microbial pathogens and environmental factors have been implicated in the pathogenesis of AAV [5].

Various potential environmental risk factors have been suggested [4, 6]. Particularly, occupational exposure to different agents has been associated with the development of AAV [7, 8]. Several case-control studies found a positive association between crystalline silica exposure and other inhaled agents with GPA and MPA [6]. Also differences in the geographic distribution have been reported suggesting that environmental factors may play a role in the pathogenesis of the disease [9]. In addition, an association with bacterial infection could be suggested in AAV [10].

Farming has been reported as a risk factor for the development of several autoimmune diseases [7, 11]. An association of farming and AAV has also been reported [12, 13]. However, there are inconsistent data about farming as a risk factor for AAV. In a recent Swedish case-control study no significantly association of farming or animal exposure with the development of AAV or GPA respectively could be detected [14]. The information about the disease and the occupation of patients in this study was obtained from registry data of inpatient care and may thus be less precise than data directly acquired using questionnaires.

The aim of the present study was to investigate in a large hospital based case-control study whether farming or farm exposure represent risk factors for the development of AAV in the north western part of Germany.

## Materials and Methods

### AAV patients and controls

The study protocol was approved by the local independent ethics committee (Ethikkommission der Ärztekammer Westfalen-Lippe). All patients and controls provided written informed consent before participating in the study. This study was conducted in compliance with Good Clinical Practices and the Declaration of Helsinki and was approved by the local independent ethics committee.

We included patients with different AAV subtypes. GPA was diagnosed according to the American College of Rheumatology (ACR) criteria and the criteria adapted from the 2012 revised Chapel Hill Consensus Conference (CHCC) [1, 15]. MPA was diagnosed according to the CHCC definition [1]. EGPA was diagnosed according to the ACR criteria and the CHCC definition [1, 16]. In addition, the EMA algorithm for classification of AAV was used to further correlate clinical and laboratory findings with the different AAV subgroups [17]. A biopsy was performed if possible. In cases in which no biopsy was available the diagnosis was defined by typical clinical manifestations and positive results for antibodies against proteinase-3 (PR3) or myeloperoxidase (MPO). ANCA-negative patients were included if a histologic confirmation of vasculitis was available.

As controls we included patients with rheumatoid arthritis (RA) and patients with large vessel vasculitis (LVV, i.e. Takayasu arteritis and giant cell arteritis) or polymyalgia rheumatica (PMR). LVV and PMR are closely related disorders [18]. Diagnosis was based on the American College of Rheumatology (ACR) 1987 criteria for RA and the criteria adapted from the 2012 revised CHCC [1, 19].

Patients and controls were selected from our university hospitals inpatient or outpatient department of Rheumatology.

In order to avoid significant differences in the geographic area of residence patients and controls were matched for the geographic catchment area at disease onset.

### Data collection, laboratory analysis

Each chart was carefully reviewed for the accuracy of the diagnosis as well as for clinical and laboratory details which includes disease onset and ANCA findings.

Disease activity of AAV was assessed by the Birmingham Vasculitis Activity Score version 3 (BVAS v.3) [20]. A relapse was defined as an increase in the disease activity. Remission was defined as an absence of clinical disease activity as indicated by a BVAS v.3 score of 0.

Carriage of MRSA was analysed in AAV patients and controls by chart review or results from nasal or pharyngeal swab culture for MRSA, respectively. In all inpatients a standardized screening for MRSA was performed at admission to our hospital (i.e. swabs from nose, throat, axilla, and groin).

Antibodies to PR3 and MPO were tested with a sensitive capture fluorescent enzyme immunoassay (Phadia EliA) performed on an ImmunoCAP 250 analyzer (Thermo Fisher Scientific, Germany).

### Questionnaire

Occupation and farm exposure was evaluated by a structured interviewer-administered questionnaire. All questionnaires were performed as direct interview either at presentation in our hospital or via telephone. The interview was performed by one and the same unblinded investigator (PW).

Patients were asked about their occupation at disease onset and during the working lifetime before. Further, a comprehensive history about farming and farming related parameters was requested. A translated version of the questionnaire can be found in the online attachment (S1 File). All questions were related either to the time of disease onset or to the time ever before (defined as > 1 year before disease onset).

The items analysed were occupation, farm exposure, participation in harvesting, exposure to cattle, pigs, horses or poultry. Our query further included current and past exposure to pets. This was performed separately for dogs, cats, and other pets. In addition subjects’ history of residence was requested. Participants were asked whether they lived on or directly next to a farm at disease onset or ever before.

Also the frequency of exposure was asked (i.e. regular defined as several times per month; occasionally defined as several times per year or rarely defined as once per year or less). All questions about exposure to different animals were related only to direct contact to these animals.

### Statistics

We compared the whole cohort of AAV patients with the control group. In addition, disease subtypes (i.e. GPA, MPA, and EGPA) were compared to the control group.

Data were analysed using SPSS20 and Medcalc 13.0 (Medcalc Software, Mariakerke, Belgium).

Univariate binary logistic regression for each exposure of interest was used to calculate odds ratios (OR), 95% confidence intervals (95%CI) and the p-values from the Wald-test.

Continuous variables were expressed as mean ± standard deviation (SD), categorical variables were summarized by absolute und relative frequencies. For categorical variables the comparisons were calculated by Fisher’s exact test or Chi-square test and for continuous variables using the Mann–Whitney U-test. The result was considered significant in case of the two-sided p < 0.05.

## Results

### Patient characteristics

**Fig 1:**
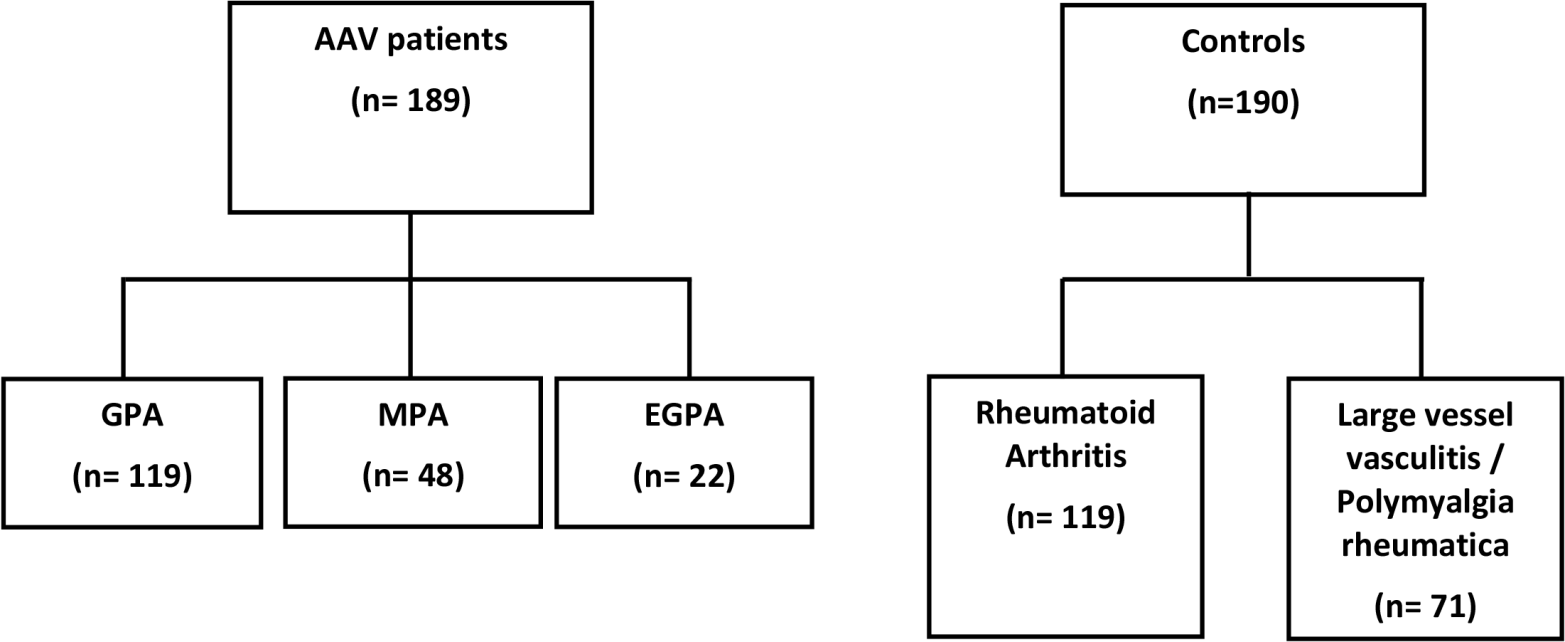
Schematic diagram showing the number of study participants in each group. AAV = ANCA associated vasculitis; GPA = granulomatosis with polyangiitis (Wegener’s); MPA = microscopic polyangiitis; EGPA = eosinophilic granulomatosis with polyangiitis.

A total of 189 patients with AAV were enrolled. In 119 patients GPA was diagnosed, 48 patients had MPA, and 22 patients suffered from EGPA. The control group consisted of 190 individuals, including 119 patients with RA and 71 patients with LVV/PMR (Fig 1). Clinical characteristics of AAV patients and controls are given in Table 1.

**Table 1:** Demographic and clinical characteristics of patients with AAV:

	AAV	*GPA*	*MPA*	*EGPA*	Control
Total	189	119	48	22	190
Age at disease onset (years)	51.2 ± 15.8	50.8 ± 16.0	54.7 ± 13.1	44.4 ± 16.4	51.6 ± 16.6
Gender (male/ female)	100 / 89	61 / 58	30 / 18	9/ 13	89 / 101
Distance to hospital (km)	46.2 ± 26.1	48.7 ± 27.9	45.4 ± 25.2	35.2 ± 22.1	46.3 ± 39.0
Biopsy performed, n (%)	163 (86.2)	101 (85)	43 (87.5)	19 (86.4)	**-**
Relapse, n (%)	93 (49.7)	68 (57.1)	16 (33.3)	9 (45)	**-**
Sinusitis, n (%)	133 (70.4)	105 (88.2)	7 (14.6)	21 (95)	**-**
Pulmonary involvement, n (%)	116 (61.4)	72 (60.5)	22 (45.8)	22 (100)	**-**
Renal involvement, n (%)	118 (63.1)	72 (60.5)	44 (91.7)	2 (10)	**-**
Polyneuropathy, n (%)	47 (24.9)	27 (22.7)	6 (12.5)	14 (63.6)	**-**
CNS Involvement, n(%)	16 (8.4)	10 (8.4)	2 (4.1)	4 (22.7)	**-**
Skin involvement, n (%)	29 (15.3)	17 (14.3)	5 (10.4)	7 (31.8)	**-**
ANCA positive (%)	163 (87.7)	115 (95.0)	47 (97.9)	3 (15)	**-**
PR3-ANCA–positive, n (%)	117 (62.6)	112 (94.1)	4 (8.3)	1 (5)	**-**
MPO-ANCA positive, n (%)	46 (24.6)	1 (0.8)	43 (89.6)	2 (10)	**-**

GPA = granulomatosis with polyangiitis (Wegener’s); MPA = microscopic polyangiitis; EGPA = eosinophilic granulomatosis with polyangiitis; MPO = myeloperoxidase; PR3 = proteinase 3; BVAS v.3 = Birmingham Vasculitis Activity Score Version 3.

There were no significant differences in age or gender between AAV patients or controls. Also we found no significant differences in the geographic area of residence between AAV patients and control groups or the distance to our hospital.

### Questionnaire

A direct interview for the questionnaire was performed in 83% of AAV patients and 80% in the control group during presentation at our hospital. In the other cases the query was performed as telephone interview. Only 2 patients considered for the control group were not willing to participate in the study. If deceased (n = 3 in the AAV group) the interview was performed by proxy (spouse or direct descendant). All other AAV patients performed the questionnaire personally.

### Occupation

Occupations of AAV patients and controls were grouped according to the following occupation-groups: students, housewives, pensioner, retailer, office workers, IT workers, industrial workers, civil servants, manual worker, farmer, miscellaneous, vehicle driver/ motor mechanic, health professional, electrician/ electrical engineer, metal worker, concrete worker, teacher, unemployed, or army/ fire department worker). We found no significant differences in the occupation groups between AAV patients and controls (S1 Fig). In particular, this held true also for main-occupation farmers.

We could not detect an increased number of occupation associated with silica exposure as suggested by Lane et al. (i.e., coal miner, coal delivery, sandblaster, mine worker, quarry worker, baker, dental worker, painting and decorating, or working in the construction industry) in the AAV compared to the control group [12]. Though, a detailed survey about qualitative and quantitative silica exposure during business or outside main occupation was not separately performed.

### Farming and farm related parameters

The questionnaire revealed that farm exposure was by no means restricted to main-occupation farmers. Indeed, from the 22 AAV patients with regular farm exposure 12 had so outside the main occupation. These patients were regularly working on a farm as additional business.

The prevalence of the different parameters analysed in the questionnaire are given in Table 2.

**Table 2:** Results of the questionnaire with prevalence for different parameters requested in AAV patients and controls at disease onset:

		AAV (%)	GPA	MPA	EGPA	Control (%)	RA	LVV/PMR
Total		189	119	48	22	190	119	71
Farm exposure		22 (11.6)	19	3	0	7 (3.68)	7	0
Residence next to farm		14 (7.41)	8	5	1	7 (3.68)	5	2
Harvesting		14 (7.41)	11	3	0	6 (2.63)	6	0
Livestock animals		27 (14.3)	23	3	1	14 (7.37)	12	2
	Cattle	16 (8.47)	15	1	0	4 (2.11)	4	0
	Pig	18 (9.52)	17	1	0	7 (3.68)	6	1
	Horse	7 (3.70)	4	3	0	6 (3.16)	5	1
	Poultry	8 (4.23)	7	0	1	9 (4.74)	7	2
Pets:		54 (28.6)	34	15	5	49 (25.8)	35	14
	Dog	39 (20.6)	27	10	2	37 (19.5)	27	10
	Cat	15 (7.93)	8	4	3	11 (5.79)	7	4
	Other	10 (5.29)	6	4	0	5 (2.63)	4	1
Ever farm exposure ^1)^		24 (12.7)	15	5	4	20 (10.5)	12	8

^1)^ = before disease onset.

GPA = granulomatosis with polyangiitis (Wegener’s); MPA = microscopic polyangiitis; EGPA = eosinophilic granulomatosis with polyangiitis; RA = rheumatoid arthritis; LVV = large vessel vasculitis; PMR = polymyalgia rheumatic.

The OR and the 95% CI are shown in Table 3. AAV as disease group was significantly associated with regular farm exposure (p = 0.005). An even higher odds ratio was found for GPA and regular farm exposure in the subgroup analysis (p< 0.001). In contrast, no significant association of MPA or EGPA with farming was found. In addition, we detected a significant association of AAV with regular contact to cattle or pigs. Again the association was restricted to GPA patients whereas no association was found for MPA or EGPA patients (Table 3). GPA was significantly associated with participation in harvesting.

**Table 3:** Odds ratio and 95% CI for different parameters of farming or animal exposure in AAV patients and controls:

		AAV vs. controls	GPA vs. controls	MPA vs. controls	EGPA vs. controls
		OR (95%CI); p	OR (95%CI); p	OR (95%CI); p	OR (95%CI); p
**Farm exposure**		3.44 (1.43–8.27); p = 0.006 *	4.97 (2.02–12.2); p< 0.001*	1.74 (0.43–7.01); p = 0.43	0.54 (0.03–9.80); p = 0.73
**Ever farm exposure**		1.24 (0.66–2.32); p = 0.51	1.22 (0.60–2.50); p = 0.58	0.99 (0.35–2.78); p = 0.98	1.98 (0.58–6.14); p = 0.21
**Harvesting**		2.45 (0.92–6.52); p = 0.07	3.12 (1.12–8.69); p = 0.029 *	2.04 (0.49–8.49); p = 0.32	0.69 (0.04–12.7); p = 0.80
**Livestock animals:**		2.01 (1.01–3.97); p = 0.046 *	3.01 (1.48–6.12); p = 0.002*	0.84 (0.23–3.04); p = 0.79	0.29 (0.02–5.10); p = 0.40
	**Cattle**	4.30 (1.41–13.1); p = 0.010 *	6.71 (2.19–20.7); p = 0.001*	0.98 (0.11–9.06); p = 0.99	1.01 (0.05–19.5); p = 0.99
	**Pig**	2.75 (1.12–6.75); p = 0.025 *	4.34 (1.75–10.9); p = 0.002 *	0.06 (0.07–4.63); p = 0.59	0.60 (0.03–10.8); p = 0.73
	**Horse**	1.17 (0.39–3.56); p = 0.78	1.06 (0.29–3.84); p = 0.92	2.04 (0.49–8.49); p = 0.33	0.69 (0.04–12.7); p = 0.80
	**Poultry**	0.89 (0.34–2.35); p = 0.81	1.26 (0.45–3.47); p = 0.66	0.19 (0.01–3.44); p = 0.26	0.46 (0.03–8.30); p = 0.60
**Pets:**		1.15 (0.73–1.81); p = 0.54	1.15 (0.69–1.92); p = 0.59	1.31 (0.65–2.61); p = 0.45	0.84 (0.29–2.41); p = 0.76
	**Dog**	1.07 (0.65–1.78); p = 0.78	1.21 (0.69–2.12); p = 0.49	1.09 (0.50–2.38); p = 0.83	0.46 (0.10–2.07); p = 0.31
	**Cat**	1.40 (0.63–3.14); p = 0.41	1.17 (0.46–3.01); p = 0.75	1.48 (0.45–4.86); p = 0.52	2.87 (0.73–11.3); p = 0.13
	**Other**	2.07 (0.69–6.17); p = 0.19	1.96 (0.59–6.59); p = 0.28	3.36 (0.87–13.0); p = 0.08	0.82 (0.04–15.4); p = 0.89
**Residence next to farm**		2.09 (0.82–5.30) p = 0.12	1.88 (0.66–5.34); p = 0.23	3.03 (0.92–10.0); p = 0.07	1.37 (0.16–11.8); p = 0.77

GPA = granulomatosis with polyangiitis; MPA = microscopic polyangiitis; EGPA = eosinophilic granulomatosis with polyangiitis; OR = odds ratio 95%CI = 95% confidence interval

* = statistically significant.

**Fig 2:**
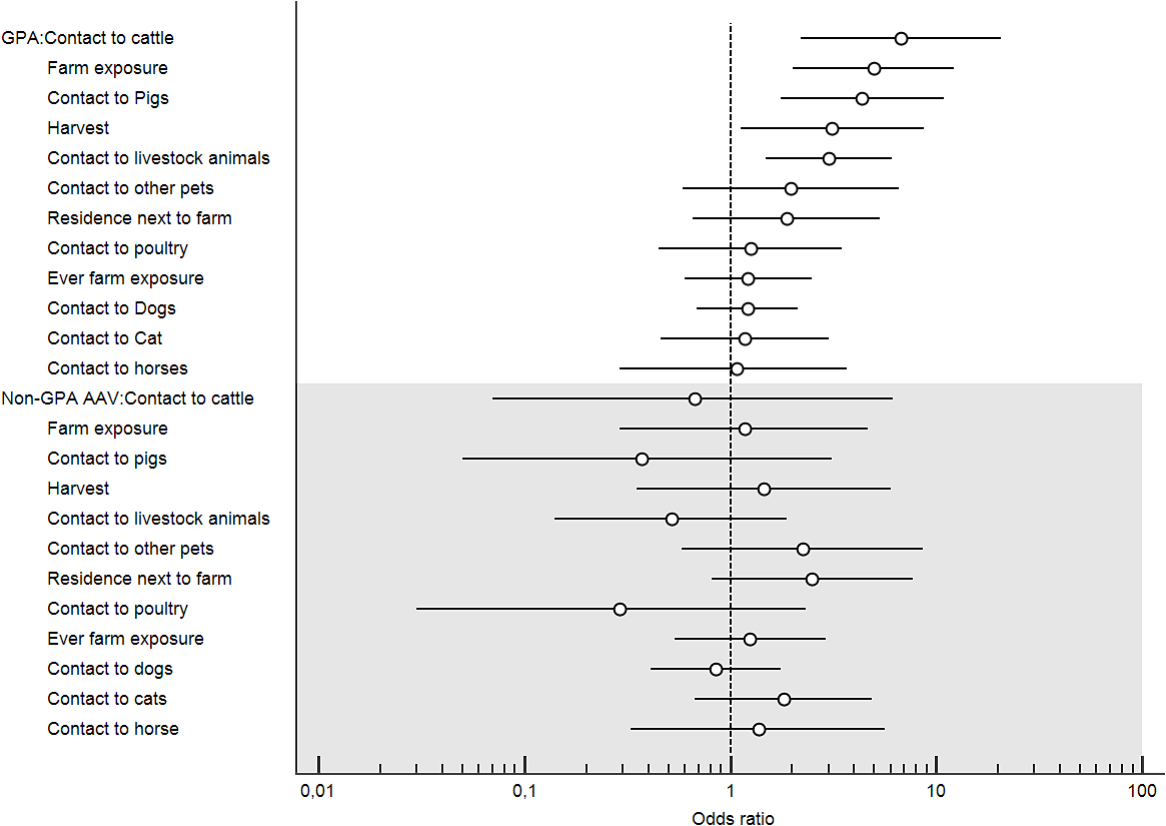
Forest Plot. The bars represent the odds ratio (circle) and 95% confidence interval for the association of GPA with different parameters (upper part) and for non-GPA patients with ANCA-associated vasculitis (lower grey part). AAV = ANCA-associated vasculitis; GPA = granulomatosis with polyangiitis (Wegener’s).

Different OR and the 95% CI of GPA patients compared to non-GPA patients with AAV (i.e. MPA; EGPA) are shown in Fig 2. The forest plot indicates high OR in patients with GPA compared to non-GPA patients with AAV in several farming related parameters. No significant associations of non-GPA patients and the items analysed in our questionnaire could be detected.

We have also evaluated the results of the questionnaire by separating patients into PR3-or MPO-ANCA positive and ANCA negative patients. The prevalence in the three patients-groups for the different items analysed were given in S1 Table. However, this analysis did not reveal an increase in the prevalence.

We could not detect significant associations of domestic pets with AAV or the subgroups.

We found no association of AAV with exposure to the different parameters ever before disease onset (). Also there was no significant difference in patients with occasional or rare frequency of farm exposure between the two groups or between the subgroups and controls (S2 Table).

Patients with farm exposure were mainly exposed to more than one type of livestock animal or other farm associated parameters analysed (i.e. participation in harvesting).

### MRSA status

MRSA status was not available in all patients (Table 4). Carriage of MRSA was tested in 164 patients with AAV (86.8%) and 121 patients in the control group (63.7%). We found an association of AAV with MRSA carriage (OR 3.38 [95%CI: 1.11–10.31]; p = 0.033). An even higher odds ratio was found in patients with GPA (OR 5.06; [95%CI: 1.62–15.78]; p = 0.005). Six out of fifteen positively tested patients had worked as farmer and had regular contact to cattle or pig (40%).

**Table 4:** Prevalence of methicillin resistant staphylococcus aureus in patients with ANCA associated vasculitis (AAV) and controls as well as odds ratio and 95% CI for MRSA in AAV patients and controls:

	AAV	GPA	MPA	EGPA	Controls	RA	LVV
Total	189	119	48	22	190	119	71
testing performed (%) ^1^	164 (86.8)	101 (84.9)	44 (91.7)	19 (86.3)	121 (63.7)	73 (61.3)	47 (66.2)
MRSA (%) ^2^	17 (10.4)	15 (14.9)	1 (2.3)	1 (5.0)	4 (3.33)	3 (4.11)	1(2.13)
	AAV vs. controls	GPA vs. controls		MPA vs. controls		EGPA vs. Controls	
OR (95%CI)	3.38 (1.11–10.33)		5.06 (1.62–15.78)		0.67 (0.07–6.20)		2.21 (0.24–20.74)
p	p = 0.032		p = 0.005		p = 0.72		p = 0.48

^1^ = number and percentage of total in each disease group.

^2^ = number and percentage of MRSA positive patients of tested patients.

GPA = granulomatosis with polyangiitis; MPA = microscopic polyangiitis; EGPA = eosinophilic granulomatosis with polyangiitis; RA = rheumatoid arthritis; LVV = large vessel vasculitis.

We found no association of MPA or EGPA with MRSA carriage compared to control. In addition, we found no significant difference in clinical manifestations between GPA patients carrying MRSA compared to GPA patients without MRSA carriage. Also no difference in the BVAS at disease onset was found (16.1 ± 7.9 vs. 17.5 ± 6.6; p = 0.27). In addition, no difference in the relapse rate was found between these groups (58.8% vs. 46.0%; p = 0.44) during the follow up period of 2.7 ± 1.5 years.

### Subgroup analysis

We found no associations with farming and a specific clinical manifestation (i.e. presence or absence of renal involvement or respiratory involvement, local manifestations) or the predominance of specific disease stages for GPA (i.e. local, early systemic, generalised or severe). One GPA patient with farm exposure was negative for PR3- and MPO-ANCAbut demonstrated a typical histology. All other GPA patients with farm exposure were positive for PR3 antibodies. AAV patients with farm exposure did not exhibit a higher BVAS at disease onset (17.4 ± 6.2 vs. 17.2 ± 6.7, p = 0.70). Also these patients did not show a significantly higher rate of relapses (63.6% vs. 49.7%, p = 0.32).

## Discussion

The pathogenesis of AAV has not yet been fully elucidated. Recent genome wide genetic association studies revealed a strong genetic link to the development of AAV [4, 5]. Our result suggests that beside the genetic contribution environmental factors may play an important role for the pathogenesis of AAV.

Since GPA, MPA, or EGPA may have different triggering factors the different entities were compared individually to the control group. Our data clearly demonstrate that the association of farming and farming related items is restricted to the GPA entity of AAV and does not involve MPA or EGPA.

The rural region surrounding our centre is characterised by a high density of livestock farming [21]. However, the control group was from the same geographic area of residence and had a significantly lower percentage of farm exposed patients. An increase of farm exposure in GPA patients due to different catchment areas could be excluded.

A possible relationship between farming and the development of AAV has been described previously [12–14]. Lane et al. detected a stronger association of farming at the time of first clinical manifestation with MPA compared to GPA. On the other hand farming during the working lifetime was significantly associated with GPA but not with MPA. However, they included only 47 patients with GPA, 12 patients with MPA, and 16 patients with EGPA. In accordance with our results they also found no association of farming with EGPA [12].

In the study of Knight et al. 2288 cases with GPA and a 10 fold larger control group was included. They found no significant association of farm-working with GPA in this large case-control study. However, the data about occupation was obtained from the Swedish Register of inpatient care and not obtained by a survey or direct interview. Occupational exposure can be different between people listed for the same occupation. Also exposure to farms or farm animals outside the occupation was not considered. In addition, the diagnosis of GPA was based on the national hospital registry referring to the discharging doctor’s medical diagnosis. This data can be incorrect to a considerable extent [14].

Also a questionnaire may not cover all aspects of farm exposure or different items associated with farming but it is much more efficient in identifying patients with farm exposure or contact to livestock animals than registry data. Several patients in our study had regular contact to farms or livestock animals during their additional business although their main occupation was not farmer or farm worker.

Lee at al. reported different clinical manifestations in GPA patients with or without high farming activity [13]. They found no association with farming in patients with localised GPA compared to patients with high farming activity who exhibited pulmonary and renal involvement in their small cohort of eight patients. We have not detected an association with different clinical manifestations in GPA patients with or without farm exposure. We could further not detect an association of farming with disease severity or the incidence of a relapse.

In accordance with the findings of Lane et al. we observed that exposure to livestock animals was stronger compared to other farm associated items (i.e. participation in harvesting) [12]. However, since most individuals were exposed to more than one item it is not possible to clearly differentiate between specific farming exposures. The strongest association of GPA was found with contact to cattle and pigs. Interestingly, we found no association of GPA with contact to horses or poultry. It is not possible to further explain this specific finding based on our data. Several factors in the care of cows or pigs may be responsible for this observation. This includes animal feeds or the use of antibiotics.

The prevalence of MRSA in GPA patients was very high. The MRSA prevalence rate (including colonisations) in Germany varies approximately from 1 to 3% [22].

We found a strong association of GPA with MRSA colonisation compared to controls. Nasal colonisation with staphylococcus aureus (staph. aureus) has been implicated in GPA pathogenesis and activity [23]. Especially the persistent colonisation of the nasal passage with these bacteria may play a pivotal role in the pathogenesis [24]. Persistence of staph. aureus colonisation is more likely in methicillin-resistant strains of this organism. It has been shown that MRSA positive farmers are more likely to be persistently colonized than transiently contaminated [25].

Staph. aureus has been described as a strong inducer of neutrophil extracellular trap (NET) formation [26]. NETs are extracellular lattices of chromatin, histones, and granule enzymes extruded by neutrophils [27]. Increased NET formation in AAV has been associated with the development of ANCA, since NETs increase the antigen exposure of PR3 or MPO to the immune system [28]. Especially chronic presence of NETs due to MRSA may facilitate stimulation of the production of ANCA [29, 30].

However, we cannot definitely substantiate whether this coincidence of MRSA carriage with GPA represents a causal relationship or not [31]. Nasal colonisation or contamination with livestock associated MRSA can be found in up to 86% of all farmers in Germany [32]. Thus, our finding of an increased MRSA colonisation can also be an epiphenomenon associated with farming.

Beside staph. aureus several other causative organisms have been associated with ANCA formation and GPA [33, 34]. Among these many bacterial pathogens are particularly common in livestock animals (i.e. campylobacter or helicobacter) [35, 36]. Thus, other livestock associated pathogens may be responsible for the association we found.

Also other factors associated with farming or the care of farm animals may play a role. An association of pesticide exposure with the development of rheumatoid arthritis or other autoimmune diseases has also been described [11]. Also an association of contact to pesticide with AAV has been reported [37]. However, this parameter was not analysed in our study. We detected an association of GPA with participation in harvesting. Perhaps crop exposure may represent a risk factor for GPA.

We found no association of silica exposure with the development of AAV. Occupational exposure to silica was not increased in AAV patients or the subgroups compared to control. An increased exposure to silica has been assumed in farmers participating in manual harvesting of potatoes, tobacco or peanuts but not in other crops [38]. Although an exposure to silica in the form of agricultural dust could not be excluded none of our patients was involved in harvesting potatoes, tobacco or peanuts. Our patients participated in grain harvesting only.

Although we could not isolate a specific trigger associated with farming we have identified farming and additional parameters associated with farming as a risk factor for AAV as a disease group, specifically in the sub group of patients with GPA. Additional structured investigations are needed to further substantiate farming as a risk factor for the development of AAV or GPA, respectively, and to identify specific determinants responsible for the pathogenesis. The results represented here may provide a concept for further analysis regarding the association of farming with the development of GPA.

## Supporting Information

S1 Fig Number of occupations at disease onset in patients with ANCA-associated vasculitis and controls. There was no significant difference in the occupation groups between ANCA-associated vasculitis patients and controls.
(TIF)

S1 File Translated version of the questionnaire. (PDF)

S1 Table Results of the questionnaire separately for patients with PR3-ANCA or MPO-ANCA and ANCA negative patients. (DOCX)

S2 Table Results of the questionnaire. (XLSX)

## References

[1] Jennette JC, Falk RJ, Bacon PA, Basu N, Cid MC, Ferrario F, et al 2012 revised International Chapel Hill Consensus Conference Nomenclature of Vasculitides. Arthritis Rheum. 2013;65: 1–11. 10.1002/art.37715 23045170

[2] Ntatsaki E, Watts RA, Scott DG. Epidemiology of ANCA-associated vasculitis. Rheum Dis Clin North Am. 2010;36: 447–461. 10.1016/j.rdc.2010.04.002 20688243

[3] Millet A, Pederzoli-Ribeil M, Guillevin L, Witko-Sarsat V, Mouthon L. Antineutrophil cytoplasmic antibody-associated vasculitides: is it time to split up the group? Ann Rheum Dis. 2013;72: 1273–1279. 10.1136/annrheumdis-2013-203255 23606701

[4] Lyons PA, Rayner TF, Trivedi S, Holle JU, Watts RA, Jayne DR, et al Genetically distinct subsets within ANCA-associated vasculitis. N Engl J Med. 2012;367: 214–223. 10.1056/NEJMoa1108735 22808956 PMC3773907

[5] Kallenberg CG, Stegeman CA, Abdulahad WH, Heeringa P. Pathogenesis of ANCA-associated vasculitis: new possibilities for intervention. Am J Kidney Dis. 2013;62: 1176–1187. 10.1053/j.ajkd.2013.05.009 23810690

[6] Mahr AD, Neogi T, Merkel PA. Epidemiology of Wegener's granulomatosis: Lessons from descriptive studies and analyses of genetic and environmental risk determinants. Clin Exp Rheumatol. 2006;24: S82–91. 16859601

[7] Miller FW, Alfredsson L, Costenbader KH, Kamen DL, Nelson LM, Norris JM, et al Epidemiology of environmental exposures and human autoimmune diseases: findings from a National Institute of Environmental Health Sciences Expert Panel Workshop. J Autoimmun. 2012;39: 259–271. 10.1016/j.jaut.2012.05.002 22739348 PMC3496812

[8] Beaudreuil S, Lasfargues G, Laueriere L, El Ghoul Z, Fourquet F, Longuet C, et al Occupational exposure in ANCA-positive patients: a case-control study. Kidney Int. 2005;67: 1961–1966. 15840044 10.1111/j.1523-1755.2005.00295.x

[9] Carruthers DM, Watts RA, Symmons DP, Scott DG. Wegener's granulomatosis—increased incidence or increased recognition? Br J Rheumatol. 1996;35: 142–145. 8612026 10.1093/rheumatology/35.2.142

[10] Chen M, Kallenberg CG. The environment, geoepidemiology and ANCA-associated vasculitides. Autoimmun Rev. 2010;9: A293–8. 10.1016/j.autrev.2009.10.008 19892038

[11] Cooper GS, Miller FW, Germolec DR. Occupational exposures and autoimmune diseases. Int Immunopharmacol. 2002;2: 303–313. 11811933 10.1016/s1567-5769(01)00181-3

[12] Lane SE, Watts RA, Bentham G, Innes NJ, Scott DG. Are environmental factors important in primary systemic vasculitis? A case-control study. Arthritis Rheum. 2003;48: 814–823. 12632437 10.1002/art.10830

[13] Lee JH, Attygalle T, Gaffney K, Scott DG. Demographics and environmental factors in a Wegener's granulomatosis cluster. Ann Rheum Dis. 2007;66: 278–279. 17242022 10.1136/ard.2006.056226 PMC1798501

[14] Knight A, Sandin S, Askling J. Occupational risk factors for Wegener's granulomatosis: a case-control study. Ann Rheum Dis. 2010;69: 737–740. 10.1136/ard.2009.107953 19364729

[15] Leavitt RY, Fauci AS, Bloch DA, Michel BA, Hunder GG, Arend WP, et al The American College of Rheumatology 1990 criteria for the classification of Wegener's granulomatosis. Arthritis Rheum. 1990;33: 1101–1107. 2202308 10.1002/art.1780330807

[16] Masi AT, Hunder GG, Lie JT, Michel BA, Bloch DA, Arend WP, et al The American College of Rheumatology 1990 criteria for the classification of Churg-Strauss syndrome (allergic granulomatosis and angiitis). Arthritis Rheum. 1990;33: 1094–1100. 2202307 10.1002/art.1780330806

[17] Watts R, Lane S, Hanslik T, Hauser T, Hellmich B, Koldingsnes W, et al Development and validation of a consensus methodology for the classification of the ANCA-associated vasculitides and polyarteritis nodosa for epidemiological studies. Ann Rheum Dis. 2007;66: 222–227. 16901958 10.1136/ard.2006.054593 PMC1798520

[18] Salvarani C, Cantini F, Hunder GG. Polymyalgia rheumatica and giant-cell arteritis. Lancet. 2008;372: 234–245. 10.1016/S0140-6736(08)61077-6 18640460

[19] Arnett FC, Edworthy SM, Bloch DA, McShane DJ, Fries JF, Cooper NS, et al The American Rheumatism Association 1987 revised criteria for the classification of rheumatoid arthritis. Arthritis Rheum. 1988;31: 315–324. 3358796 10.1002/art.1780310302

[20] Mukhtyar C, Lee R, Brown D, Carruthers D, Dasgupta B, Dubey S, et al Modification and validation of the Birmingham Vasculitis Activity Score (version 3). Ann Rheum Dis. 2009;68: 1827–1832. 10.1136/ard.2008.101279 19054820

[21] Kock R, Schaumburg F, Mellmann A, Koksal M, Jurke A, Becker K, et al Livestock-associated methicillin-resistant Staphylococcus aureus (MRSA) as causes of human infection and colonization in Germany. PLoS One. 2013;8: e55040 10.1371/journal.pone.0055040 23418434 PMC3572123

[22] Kock R, Mellmann A, Schaumburg F, Friedrich AW, Kipp F, Becker K. The epidemiology of methicillin-resistant Staphylococcus aureus (MRSA) in Germany. Dtsch Arztebl Int. 2011;108: 761–767. 10.3238/arztebl.2011.0761 22163252 PMC3230165

[23] Laudien M, Gadola SD, Podschun R, Hedderich J, Paulsen J, Reinhold-Keller E, et al Nasal carriage of Staphylococcus aureus and endonasal activity in Wegener s granulomatosis as compared to rheumatoid arthritis and chronic Rhinosinusitis with nasal polyps. Clin Exp Rheumatol. 2010;28: 51–55. 20412703

[24] Lutalo PM, D'Cruz DP. Diagnosis and classification of granulomatosis with polyangiitis (aka Wegener's granulomatosis). J Autoimmun. 2014;48–49: 94–98. 10.1016/j.jaut.2014.01.028 24485158

[25] Kock R, Loth B, Koksal M, Schulte-Wulwer J, Harlizius J, Friedrich AW. Persistence of nasal colonization with livestock-associated methicillin-resistant Staphylococcus aureus in pig farmers after holidays from pig exposure. Appl Environ Microbiol. 2012;78: 4046–4047. 10.1128/AEM.00212-12 22447613 PMC3346418

[26] Pilsczek FH, Salina D, Poon KK, Fahey C, Yipp BG, Sibley CD, et al A novel mechanism of rapid nuclear neutrophil extracellular trap formation in response to Staphylococcus aureus. J Immunol. 2010;185: 7413–7425. 10.4049/jimmunol.1000675 21098229

[27] Rondina MT, Weyrich AS, Zimmerman GA. Platelets as cellular effectors of inflammation in vascular diseases. Circ Res. 2013;112: 1506–1519. 10.1161/CIRCRESAHA.113.300512 23704217 PMC3738064

[28] Ohlsson SM, Ohlsson S, Soderberg D, Gunnarsson L, Pettersson A, Segelmark M, et al Neutrophils from vasculitis patients exhibit an increased propensity for activation by anti-neutrophil cytoplasmic antibodies. Clin Exp Immunol. 2014;. 10.1111/cei.12555 24666336 PMC4008980

[29] Lögters T, Margraf S, Altrichter J, Cinatl J, Mitzner S, Windolf J, et al The clinical value of neutrophil extracellular traps. Med Microbiol Immunol (Berl). 2009;198: 211–219. 19653000 10.1007/s00430-009-0121-x

[30] Berends ET, Horswill AR, Haste NM, Monestier M, Nizet V, von Kockritz-Blickwede M. Nuclease expression by Staphylococcus aureus facilitates escape from neutrophil extracellular traps. J Innate Immun. 2010;2: 576–586. 10.1159/000319909 20829609 PMC2982853

[31] Mutneja R, Shah M, Datta D. Methicillin Resistant Staphylococcus Aureus (MRSA) and Granulomatosis With Polyangiitis: Coincidence or a Causal Relationship? CHEST Journal. 2013;144: 354A–354A.

[32] Cuny C, Nathaus R, Layer F, Strommenger B, Altmann D, Witte W. Nasal colonization of humans with methicillin-resistant Staphylococcus aureus (MRSA) CC398 with and without exposure to pigs. PLoS One. 2009;4: e6800 10.1371/journal.pone.0006800 19710922 PMC2728842

[33] Konstantinov KN, Ulff-Moller CJ, Tzamaloukas AH. Infections and antineutrophil cytoplasmic antibodies: Triggering mechanisms. Autoimmun Rev. 2015;14: 201–203. 10.1016/j.autrev.2014.10.020 25448042

[34] Lidar M, Lipschitz N, Langevitz P, Barzilai O, Ram M, Porat-Katz BS, et al Infectious serologies and autoantibodies in Wegener's granulomatosis and other vasculitides: novel associations disclosed using the Rad BioPlex 2200. Ann N Y Acad Sci. 2009;1173: 649–657. 10.1111/j.1749-6632.2009.04641.x 19758211

[35] Oporto B, Esteban JI, Aduriz G, Juste RA, Hurtado A. Prevalence and strain diversity of thermophilic campylobacters in cattle, sheep and swine farms. J Appl Microbiol. 2007;103: 977–984. 17897201 10.1111/j.1365-2672.2007.03328.x

[36] Dimola S, Caruso ML. Helicobacter pylori in animals affecting the human habitat through the food chain. Anticancer Res. 1999;19: 3889–3894. 10628327

[37] Duna GF, Cotch MF, Galperin C, Hoffman DB, Hoffman GS. Wegener's granulomatosis: role of environmental exposures. Clin Exp Rheumatol. 1998;16: 669–674. 9844758

[38] Hogan SL, Cooper GS, Savitz DA, Nylander-French LA, Parks CG, Chin H, et al Association of silica exposure with anti-neutrophil cytoplasmic autoantibody small-vessel vasculitis: a population-based, case-control study. Clin J Am Soc Nephrol. 2007;2: 290–299. 17699427 10.2215/CJN.03501006 PMC4049534

