# Supplementary figures and images for: Farm Exposure as a Differential Risk Factor in ANCA-Associated Vasculitis
Source: PLoS One. 2015 Sep 4;10(9):e0137196. doi: 10.1371/journal.pone.0137196 (PMC4560371; doi:10.1371/journal.pone.0137196)

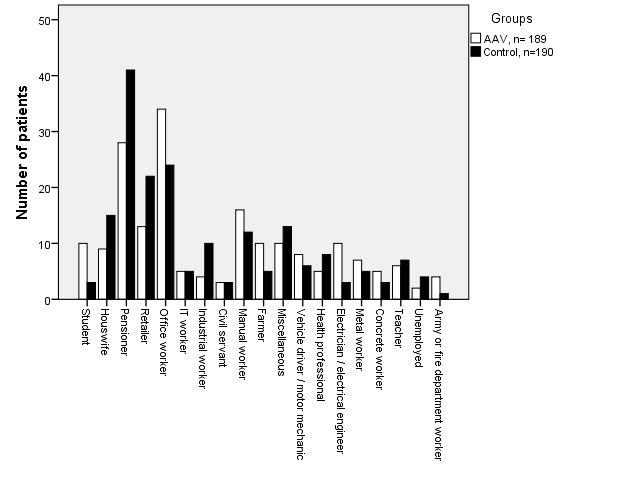

Supplement: S1 Fig — There was no significant difference in the occupation groups between ANCA-associated vasculitis patients and controls. (TIF) [file pone.0137196.s001.tif]
