# Supplementary material for: Farm Exposure as a Differential Risk Factor in ANCA-Associated Vasculitis
Source: PLoS One. 2015 Sep 4;10(9):e0137196. doi: 10.1371/journal.pone.0137196 (PMC4560371; doi:10.1371/journal.pone.0137196)
Supplement: S1 File — (PDF) [file pone.0137196.s002.pdf]

**Questionnaire:**

1. What was your occupation at disease onset? ________________________________

   ______________________________________________________________________

2. If different, occupation before disease onset:

   ______________________________________________________________________

3. Are you living or working on a farm: ☐ yes ☐ no

   If yes, since when (year)? from: _____________ to: __________

Additional comments: ______________________________________________________

4. Did you ever live or work on a farm: ☐ yes ☐ no

   If yes, since when (year)? from: _____________ to: __________

Additional comments: ______________________________________________________

5. Contact to livestock animals? ☐ yes ☐ no

   a. If yes, contact Cows / cattle: from: _____________ to: __________

      Pigs: from: _____________ to: __________

      Horses: from: _____________ to: __________

      Poultry: from: _____________ to: __________

   b. Frequency of contact: ☐ Regular (several times per month):

      ☐ Occasionally (several times per year):

      ☐ Rarely (once per year or less):

Additional comments: ______________________________________________________

6. Do you participate in harvesting? ☐ yes ☐ no

If yes, since when (year)? from: _____________ to: __________

What do or did you harvest? ____________________

Additional comments: ____________________________________________________

7. Are you living or did you ever life next to a farm? ☐ yes ☐ no

   a. If yes, since when (year)? from: _____________ to: __________

   b. If yes, estimated distance: _____________ meters

Additional comments: ____________________________________________________

8. Do you or did you have regular contact to pets: ☐ yes ☐ no

   a. If yes, since when (year)? from: _____________ to: __________

   b. What kind of pet (dog, cat,…) _______________________________

Additional comments: ____________________________________________________

9. Further comments:

____________________________________________________________________

____________________________________________________________________

____________________________________________________________________
