# Supplementary material for: Farm Exposure as a Differential Risk Factor in ANCA-Associated Vasculitis
Source: PLoS One. 2015 Sep 4;10(9):e0137196. doi: 10.1371/journal.pone.0137196 (PMC4560371; doi:10.1371/journal.pone.0137196)
Supplement: S1 Table — (DOCX) [file pone.0137196.s003.docx]

**S1 Table:** Results of the questionnaire separately for patients with PR3-ANCA or MPO-ANCA and ANCA negative patients.

|  |  | **AAV (%)** | PR3-ANCA | MPO-ANCA | no ANCA |
| --- | --- | --- | --- | --- | --- |
| Total |  | 189 | 117 | 46 | 26 |
| Farm exposure | | 22 (11.6) | 17 | 4 | 1 |
| Residence next to farm | | 14 (7.41) | 7 | 5 | 2 |
| Harvesting |  | 14 (7.41) | 10 | 4 | 0 |
| Livestock animals | | 27 (14.3) | 20 | 4 | 3 |
|  | Cattle | 16 (8.47) | 14 | 1 | 1 |
|  | Pig | 18 (9.52) | 16 | 1 | 1 |
|  | Horse | 7 (3.70) | 2 | 3 | 2 |
|  | Poultry | 8 (4.23) | 5 | 1 | 2 |
| Pets: |  | 54 (28.6) | 33 | 14 | 7 |
|  | Dog | 39 (20.6) | 26 | 10 | 3 |
|  | Cat | 15 (7.93) | 8 | 3 | 4 |
|  | Other | 10 (5.29) | 6 | 4 | 0 |
| Ever farm exposure ^1)^ | | 24 (12.7) | 15 | 6 | 3 |

^1)^= before disease onset
